# An Examination of Chemical Tools for Hydrogen Selenide Donation and Detection

**DOI:** 10.3390/molecules29163863

**Published:** 2024-08-15

**Authors:** Rynne A. Hankins, John C. Lukesh

**Affiliations:** Department of Chemistry, Wake Forest University, Wake Downtown Campus, 455 Vine Street, Winston-Salem, NC 27101, USA

**Keywords:** gasotransmitters, signaling, donors, sensors, hydrogen selenide, H_2_Se

## Abstract

Hydrogen selenide (H_2_Se) is an emerging biomolecule of interest with similar properties to that of other gaseous signaling molecules (i.e., gasotransmitters that include nitric oxide, carbon monoxide, and hydrogen sulfide). H_2_Se is enzymatically generated in humans where it serves as a key metabolic intermediate in the production of selenoproteins and other selenium-containing biomolecules. However, beyond its participation in biosynthetic pathways, its involvement in cellular signaling or other biological mechanisms remains unclear. To uncover its true biological significance, H_2_Se-specific chemical tools capable of functioning under physiological conditions are required but lacking in comparison to those that exist for other gasotransmitters. Recently, researchers have begun to fill this unmet need by developing new H_2_Se-releasing compounds, along with pioneering methods for selenide detection and quantification. In combination, the chemical tools highlighted in this review have the potential to spark groundbreaking explorations into the chemical biology of H_2_Se, which may lead to its branding as the fourth official gasotransmitter.

## 1. Introduction

The discovery of elemental selenium can be traced back to a Swedish sulfuric acid plant in the early 19th Century where chemist Jöns Jakob Berzelius first observed a reddish-brown sediment in the acid being produced there [1]. Berzelius initially mistook this new substance for tellurium due to its odor and appearance. Famed for his advancement of modern chemical notation, the principle of stoichiometry, and the determination of atomic weights of most known elements at the time, Berzelius chemically compared the red-brown byproduct with a known sample of tellurium and determined that the two were, in fact, different elements with the new substance having the properties of a metal combined with that of sulfur. In the words of Berzelius, he had discovered “a new kind of sulfur”.

Today, selenium is regarded as an essential micronutrient that is acquired through dietary means with an optimal daily dose of 55 µg for adults [2]. There are at least 25 selenoproteins [3,4,5], many of which play a central role in cellular redox homeostasis, including glutathione peroxidases (GPx) and thioredoxin reductases (TrxR), and require dietary selenium for their production. Low selenium levels in humans are associated with a myriad of illnesses [6,7,8,9]. Keshan Disease [10,11], a potentially fatal form of cardiomyopathy, is primarily observed in selenium-deficient regions in China. The same is true for Kashin–Beck Disease [12,13], a chronic joint disease predominantly found in parts of the world where selenium is scarce. Weakened immune function [14,15], cardiovascular diseases [16,17], and certain cancers [18,19,20,21] are also strongly associated with selenium deficiency. These correlative studies suggest that the role of selenium in human health and biology could extend beyond its incorporation into selenium-containing proteins and point towards its possible involvement in other cellular processes. 

In terms of its chemistry, selenium, like sulfur, can exist in numerous oxidation states, including selenate (SeO_4_^2−^, +6), selenite (SeO_3_^2−^, +4), and selenide (Se^2−^, −2) [22]. Selenide, the most reduced form of selenium, is the heavier chalcogen counterpart to sulfide (S^2−^). Both sulfide and selenide exist in different protonation states, dependent on environmental pH, with the fully protonated forms of both—hydrogen sulfide (H_2_S, p*K*_a1_: 6.9) [23] and hydrogen selenide (H_2_Se, p*K*_a1_: 3.9) [24]—being formerly dismissed as highly toxic gases with little biological relevance [25,26]. However, in the case of hydrogen sulfide, this malodorous gas has recently experienced a rebirth as an important, endogenous signaling molecule (or gasotransmitter) in mammals [27,28,29,30]. 

H_2_S is primarily produced by three principal enzymes—cystathionine β-synthase (CBS) [31], cystathionine *γ*-lyase (CSE) [32], and 3-mercaptopyruvate sulfur transferase (3-MST) [33]—providing exquisite control over its production. As such, endogenous H_2_S is known to be involved in numerous signaling processes throughout the body, including the brain and central nervous system [34,35], and within specific cellular compartments (i.e., mitochondria) [36,37]. Much of what is known about H_2_S pharmacology stems from the advent of donor compounds, synthetic small molecules designed to slowly liberate H_2_S in a controlled fashion that mimics its natural biosynthesis, and the use of said compounds in various cellular and animal models of disease [38,39,40,41].

Alongside nitric oxide (NO) and carbon monoxide (CO), hydrogen sulfide is the most recent, widely recognized member of the gasotransmitter family [42,43,44]. Its inclusion was suggested in the early 2000s and was based on five key observations [45]. Namely, H_2_S is (i) a small molecule gas that (ii) freely permeates cellular membranes. It is (iii) endogenously and enzymatically generated, with (iv) well-defined biological functions that stem from its (v) action at specific cellular targets. 

**Figure 1:**
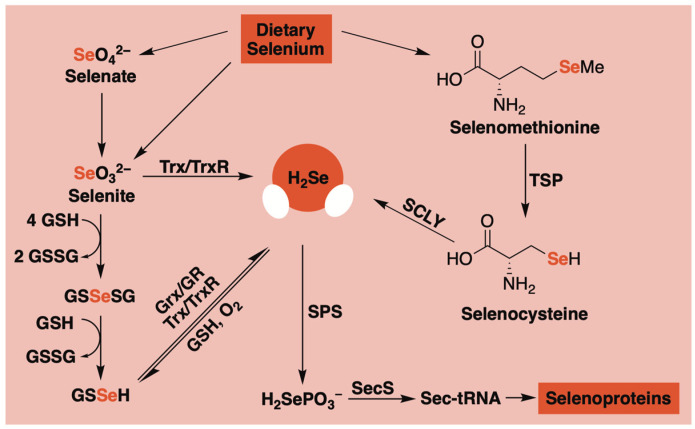
A simplified schematic of H_2_Se production in mammalian systems. Glutathione (GSH), glutathione disulfide (GSSG), glutaredoxin (Grx), glutathione reductase (GR), thioredoxin (Trx), thioredoxin reductase (TrxR), transsulfuration pathway (TSP), selenocysteine lyase (SCLY), selenophosphate synthetase (SPS), and selenocysteine synthase (SecS).

It is interesting to note that H_2_Se (Predominantly HSe^−^ at physiological pH) already checks several of these boxes. It exists as a lipophilic gas in its diprotic form and is expressed enzymatically in mammals where it serves as a key intermediate in the production of selenium-containing biomolecules (Figure 1) [46]. H_2_Se was shown to act on at least some protein targets, including inhibition of cytochrome c oxidase, which modulates aerobic respiration [46,47]. Moreover, it is the reduced selenide form of selenium, and not the often administrated selenite (or other oxidized forms), that is believed to be responsible for the observed biological activity of selenium, including its anticancer effects and noted protection against myocardial ischemia reperfusion injury [48,49,50]. 

Still, compared to H_2_S, very little is known about the (patho)physiological effects of H_2_Se. This is due, at least in part, to a lack of refined donor compounds that can increase the bioavailability of selenide and be used to effectively modulate cellular concentrations. We and others have begun to fill this unmet need by developing new H_2_Se-releasing compounds, which we will highlight below, along with current methods for selenide detection and quantification. In combination, the chemical tools outlined in this review have the potential to serve as invaluable exploratory tools for uncovering H_2_Se biology and its potential in medicine. 

## 2. Chemical Tools for H_2_Se Donation

### 2.1. Selenotrisulfides

**Figure 2:**
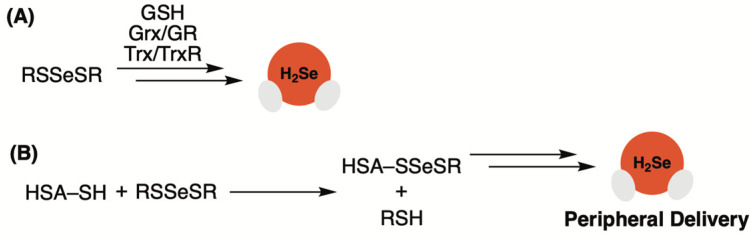
(**A**) Selenotrisulfides as H_2_Se donors. (**B**) Peripheral delivery of H_2_Se via human serum albumin. Glutathione (GSH), glutaredoxin (Grx), glutathione reductase (GR), thioredoxin (Trx), thioredoxin reductase (TrxR), and human serum albumin (HSA).

A key intermediate in the biosynthesis of H_2_Se is believed to be glutathione selenotrisulfide (GSSeSG), which forms in vitro from the reduction of selenite with four equivalents of glutathione (GSH) [51,52,53]. While the isolation of GSSeSG is difficult due to its chemical instability, the reduction of selenite is not specific to GSH, meaning the preparation and evaluation of alternative selenotrisulfides (RSSeSR) as potential H_2_Se-donating motifs could be a viable option (Figure 2A). 

In a pioneering study by Nakayama and co-workers, the more robust penicillamine selenotrisulfide (PenSSeSPen) was prepared, which is isolable due to increased steric bulk near the S–Se–S motif, and its bioavailability was compared with that of selenite, an established dietary source of selenium, in Se-deficient mice [54]. Following oral administration, the selenium content in selected organs was quantified fluorometrically using 2,3-diaminonaphthalene. Similar to selenite-treated mice, the administration of PenSSeSPen led to a significant increase in selenium in the heart, liver and blood. Additionally, PenSSeSPen-fed mice exhibited similar GPx activity to that of selenite-fed mice, indicating that the selenium content from PenSSeSPen was available for selenoprotein production.

In a later study by Nakayama, it was proposed that human serum albumin (HSA), the most abundant plasma protein, serves as a selenium carrier via a selenotrisulfide linkage that enables it to distribute selenide to peripheral tissues and organs throughout the body [55]. The authors observed that treatment of red blood cells (RBCs) with selenite led to selenium efflux that was dependent on HSA concentration. Moreover, pretreatment of HSA with iodoacetamide, a thiol-blocking agent, appeared to inhibit selenium transfer from RBCs to HSA, confirming that the thiol functional group on HSA played a key role in the transfer event. When selenium-bound HSA was treated with penicillamine (Pen), selenotrisulfide PenSSeSPen was produced. Additionally, the same PenSSeSPen product was formed when the selenium efflux experiment was conducted in the presence of Pen rather than HSA. These observations led to the conclusion that selenium is likely to be exported from RBCs as a selenotrisulfide (RSSeSR), which forms from the reaction between selenite and an RBC thiol. The ensuing selenotrisulfide then binds to HSA via a thiol exchange reaction, enabling the transport of selenium throughout the body (Figure 2B). 

In a very recent study by Wang, Xu, Xie, and co-workers, it was reported that a stable selenotrisulfide (AcidSSeSAcid) could be formed by treating 2-mercaptoacetic acid with selenium dioxide [56]. However, when the same reaction was run with 2-mercaptoethanol, the resultant selenotrisulfide (HydSSeSHyd) behaved more like GSSeSG and proved difficult to characterize. Thus, only the reactivity of the more robust AcidSSeSAcid was assessed further in the presence of glutathione. Using ESI-MS, the byproducts of this reaction were found to be GSSG, AcidSH, AcidSSeSG, AcidSSG, and AcidSSeH, providing some indirect evidence of glutathione-promoted H_2_Se release. At the outset of this study, the authors hypothesized that H_2_Se might function as an “H_2_S disguiser” and thereby assist in overcoming H_2_S-indcued antibiotic resistance. This theory was tested with AcidSSeSAcid in an H_2_S-induced antibiotic-resistant MRSA model (MRSA^S+^). While the antibiotic gentamicin alone proved ineffective against MRSA^S+^ it, in combination with AcidSSeSAcid, displayed impressive bactericidal activity. The authors attributed this reduction in antibiotic resistance to the release of H_2_Se, which increases bacterial membrane permeability and reactivates bacterial respiratory flux. 

Although the direct liberation of H_2_Se was not confirmed in any of these studies with trapping experiments, they do underscore the biological relevance of the RSSeSR motif and its likely ability to function as an endogenous selenide delivery agent. The general instability of this framework, however, may limit its overall utility as an exogenous source of H_2_Se, as the ability to generate a large library of selenotrisulfides with tunable rates of release could prove difficult. Thus, the search continues for alternative frameworks with the potential to supply selenide in a controlled and sustained fashion. 

### 2.2. Selenide Salts

Logically, many H_2_Se donors were inspired by previously reported H_2_S-releasing compounds. To this end, selenide salts, which serve as a convenient H_2_Se equivalent in buffer, were examined for convenience [47,50,57], drawing parallels to sulfide salts being used in initial studies aimed at exploring H_2_S chemical biology [58].

Using sodium hydroselenide (NaHSe), the Dyson group was among the first to explore in detail the pharmacology and therapeutic utility of H_2_Se [47]. Given its instability and absence of a reliable commercial source, the group elected to generate NaHSe in-house by reducing elemental selenium with an aqueous solution of sodium borohydride [59]. Once in hand, they evaluated the metabolic effects of NaHSe ex vivo using dissected rat soleus muscle and homogenized liver tissue. In these models, NaHSe was shown to inhibit oxygen consumption in a concentration-dependent manner, albeit to a lesser extent than sodium hydrosulfide (NaHS) and potassium cyanide (KCN), which were used as positive controls. The authors also investigated the mechanism of inhibition of O_2_ consumption and found its inhibition of cytochrome C oxidase to be a likely candidate, similar to NaHS.

The influence of NaHSe on selenoprotein expression in HepG2 (human hepatocyte) cells was also inspected [47]. Using a Western blot analysis, a significant increase in the production of glutathione peroxidase-1 (GPx-1) was observed upon treatment with NaHSe. Interestingly, the addition of selenite (SeO_3_^2−^), a common dietary source of selenium, had the opposite effect with a notable reduction in GPx-1 expression compared to nontreated controls. Moreover, the Dyson group also demonstrated that the addition of DL-propargylglycine (PAG), an established CSE inhibitor, led to significant reductions in both GPx-1 and thioredoxin reductase-1 (TrxR), presumably due to its inhibition of the H_2_Se producing enzyme selenocysteine lyase (SCLY, Figure 1). However, the expression of both proteins was restored by the addition of exogenous NaHSe, suggesting that endogenous selenoprotein expression can be regulated by H_2_Se administration.

The therapeutic value of H_2_Se supplementation was also examined in HepG2 cells exposed to hydrogen peroxide (H_2_O_2_) insult [47]. Indeed, incubation with NaHSe (0.3 and 1 µM) for 1 h conferred cellular protection in a dose-dependent manner. The authors noted that multiple mechanisms could be in play to account for the improved ROS management by NaHSe-treated cells, including its direct ROS scavenging, its functioning as a metabolic modulator, and/or its serving as the catalytic component of antioxidant selenoproteins. 

Finally, the in vivo pharmacological effects of NaHSe were also investigated in this study [47]. Anesthetized rats were given an escalated dose of 0.01 mg/kg to 10 mg/kg of selenide. Both blood pressure and heart rate decreased notably at the highest dose level but, overall, cardiac output remained relatively unaffected. The authors also noted significant hyperlactatemia (inhibition of oxidative phosphorylation) at the highest dose of NaHSe.

To date, this remains one of the most comprehensive studies, detailing the chemical biology and pharmacological effects of H_2_Se [47]. Still, a severe limitation, even noted by the authors, was the use of NaHSe as an H_2_Se source. As cited above, the use of selenide salts is analogous to early work on H_2_S which employed sulfide salts as a convenient method for H_2_S delivery. Their use, however, creates a bolus effect that poorly mimics the endogenic production of H_2_S. The same is likely true for the use of selenide salts as a research tool for examining the pharmacology and medicinal value of H_2_Se. Thus, refined selenium-containing compounds with suitable pharmacokinetics and exquisite control over their selenide release are highly desirable and early attempts to access such compounds are summarized below. 

### 2.3. Selenoanhydrides

**Figure 3:**
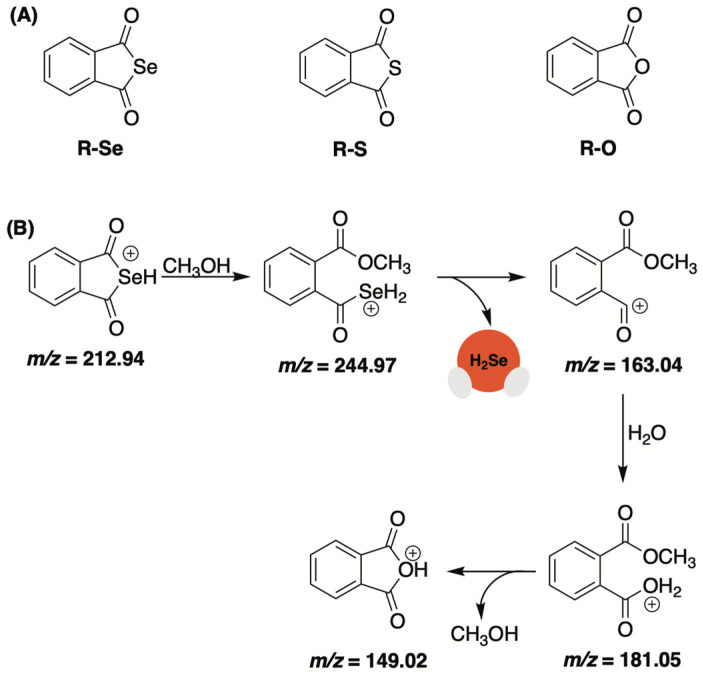
(**A**) Chalcogen anhydrides (R-Se, R-S, and R-O) under examination; (**B**) Proposed fragmentation pattern for R-Se in 50% methanol/water under ESI conditions.

Selenoanhydrides were among the first small molecules assessed for their ability to deliver selenide in a controlled fashion and in response to biologically pertinent molecules [60]. Domínguez-Álvarez and co-workers reported on the impressive anticancer effects of selenoanhydride R-Se (Figure 3A) in earlier studies and suspected it might be due to its release of H_2_Se [61,62,63]. To examine this, they monitored the fragmentation pattern of R-Se in a 50% methanol/water solution. Under electrospray ionization conditions (ESI), initial attack by methanol led to the observed fragmentation products with the loss of H_2_Se (Figure 3B). Moreover, the addition of Na_2_S appeared to amplify the decomposition of the donor with selenium-containing fragments being observed but with a significantly lower abundance. While experimental conditions were not biologically relevant, this simple ESI-MS analysis did provide some insight into the propensity of selenoanhydrides to expel selenide upon nucleophilic exposure.

The reducing capacity of R-Se and related chalcogens (R-S and R-O, Figure 3A) was assessed using cPTIO, a nitric oxide radical scavenger [60]. The authors noted that the addition of H_2_S potentiated the radical scavenging ability of R-Se and R-S (albeit to a lesser extent) but not R-O. The same trend was observed with the antioxidant glutathione (GSH). While the addition of GSH alone did not effectively quench cPTIO, it in combination with R-Se was found to significantly reduce cPTIO radicals. Similar results were obtained with superoxide (O_2_^•−^). Using BMPO as an EPR spin trap reagent, the authors found that H_2_S/R-Se and H_2_S/R-S (but not H_2_S/R-O) could effectively scavenge BMPO-OOH/OH adducts and, perhaps, O_2_^•−^ directly. 

In total, these observations indicate that the reducing potency of thiols (i.e., GSH and H_2_S) are significantly boosted upon their interaction with R-Se (and to a lesser extent R-S), which is likely to liberate reactive selenium (or sulfur) species, including hydrogen selenide. Although these studies never provided any direct evidence of H_2_Se release from R-Se, the radical scavenging activity of this purported donor provides a strong indication. 

### 2.4. P=Se Motifs

**Figure 4:**
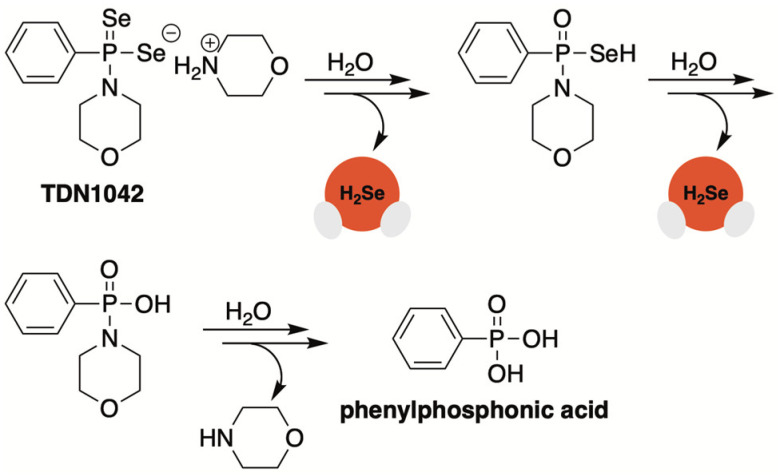
Proposed hydrolytic pathway of TDN1042, which results in the release of two equivalents of H_2_Se and the formation of phenylphosphonic acid.

With inspiration from GYY4137, an early and extensively studied H_2_S donor that gradually decomposes to release H_2_S via P=S bond cleavage [64], Pluth and co-workers developed an analogous H_2_Se donor, TDN1042, that delivered selenide (H_2_S/H_2_Se^−^) via a similar hydrolytic pathway (Figure 4) [65]. Synthetically, TD1042 was accessed upon treatment of Woollins’ reagent with excess morpholine, akin to the preparation of GYY4137. 

Once prepared and structurally characterized by NMR and single crystal X-ray diffraction, H_2_Se release from TDN1042 in wet DMSO was evaluated using ^31^P NMR spectroscopy. These studies revealed the clean conversation of TD1042 to phenylphosphonic acid, as expected (Figure 4). This clean transformation to phenylphosphonic acid was also observed in citrate buffer (pH 3.0 to 6.0), with higher rates of release occurring at more acidic pH values, which is consistent with a hydrolysis-based mechanism. 

The authors also confirmed the direct liberation of H_2_Se from TD1042, a key experiment that was omitted from previous reports of H_2_Se donating motifs. To accomplish this, an aqueous solution of TD1042 was acidified with HCl and sparged with N_2_ to assist in volatilizing any released H_2_Se into the vial headspace where it was then bubbled through a separate trapping solution of dinitrofluorobenzene (DNFB). Using HPLC, Pluth and co-workers observed the formation of both di(2,4-dinitrophenyl) selenide ((DNP)_2_Se) and the related diselenide ((DNP)_2_Se_2_) in the trapping solution. 

**Figure 5:**
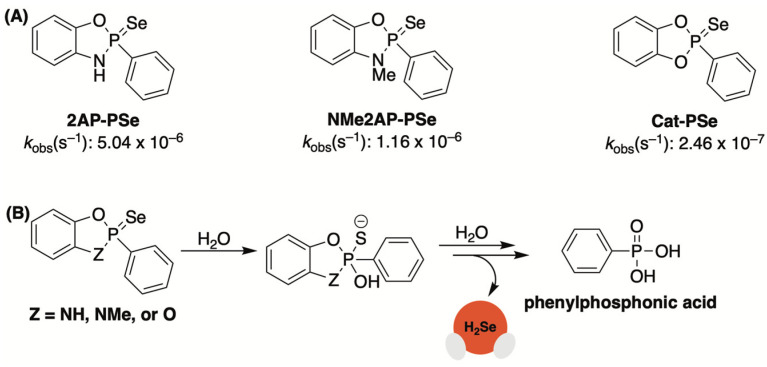
(**A**) Cyclic-PSe donors arranged in order of decreasing rates of hydrolysis; (**B**) Proposed hydrolytic pathway of cyclic-PSe donors, which results in the release of one equivalent of H_2_Se and the formation of phenylphosphonic acid.

In a later study, the Pluth group sought to augment the rate of H_2_Se donation from this platform through the introduction of cyclic-PSe donors [66]. To accomplish this, Woollins’ reagent was treated with various *ortho*-substituted phenols to generate a small library of donors with a single P=Se motif (Cat-PSe, 2AP-PSe, and NMe2AP-PSe, Figure 5A). 

By introducing a second electronegative heteroatom (i.e., oxygen or nitrogen), it was suspected that the rate of hydrolysis would increase due to the enhanced electrophilic character of the phosphorous center. This was confirmed by ^31^P NMR, which was used to monitor donor hydrolysis in PIPES buffer (pH 7.4). Like TD1042, cyclic-PSe compounds were found to release selenide while cleanly forming phenylphosphonic acid as a byproduct (Figure 5B). However, unlike TD1042, these donors were shown to operate at neutral pH due to their enhanced hydrolytic lability. The rates of hydrolysis among cyclic-PSe donors varied significantly with Cat-PSe displaying the slowest rate of hydrolysis and 2AP-PSe hydrolyzing the quickest. This result was somewhat unexpected and suggests that factors other than electronic effects influence the rates of hydrolysis. 

Cell permeability studies were also conducted using time-of-flight secondary ion mass spectrometry (TOF-SIMS). A dose-dependent increase in intracellular selenium levels was observed in HeLa cells with increasing concentrations of 2AP-PSe, the most efficient H_2_Se donor identified in this study.

The in cellulo antioxidant activity of 2AP-PSe was also assessed. When live HeLa cells were treated with exogenous H_2_O_2_ (500 µM) and peroxide sensor DCFH-DA, a significant fluorescence response was observed due to the formation of 2′,7′-dichlorofluorescein (DCF) [67]. However, when cells were first pre-treated with 2AP-PSe (5–25 µM) a notable decrease in ROS-generated fluorescence was observed. Due to the instability of H_2_Se, the authors stress that the observed effects are unlikely due to the buildup of H_2_Se, but rather an increase in selenocompounds (likely antioxidant proteins) that effectively scavenge ROS. 

### 2.5. Selenocarbonyls

In a comprehensive study by Yi and co-workers, both selenocyclopropenones and arylselenoamides were found to provide highly tunable rates of H_2_Se delivery in the presence of supraphysiological concentrations of cysteine [68]. 

**Figure 6:**
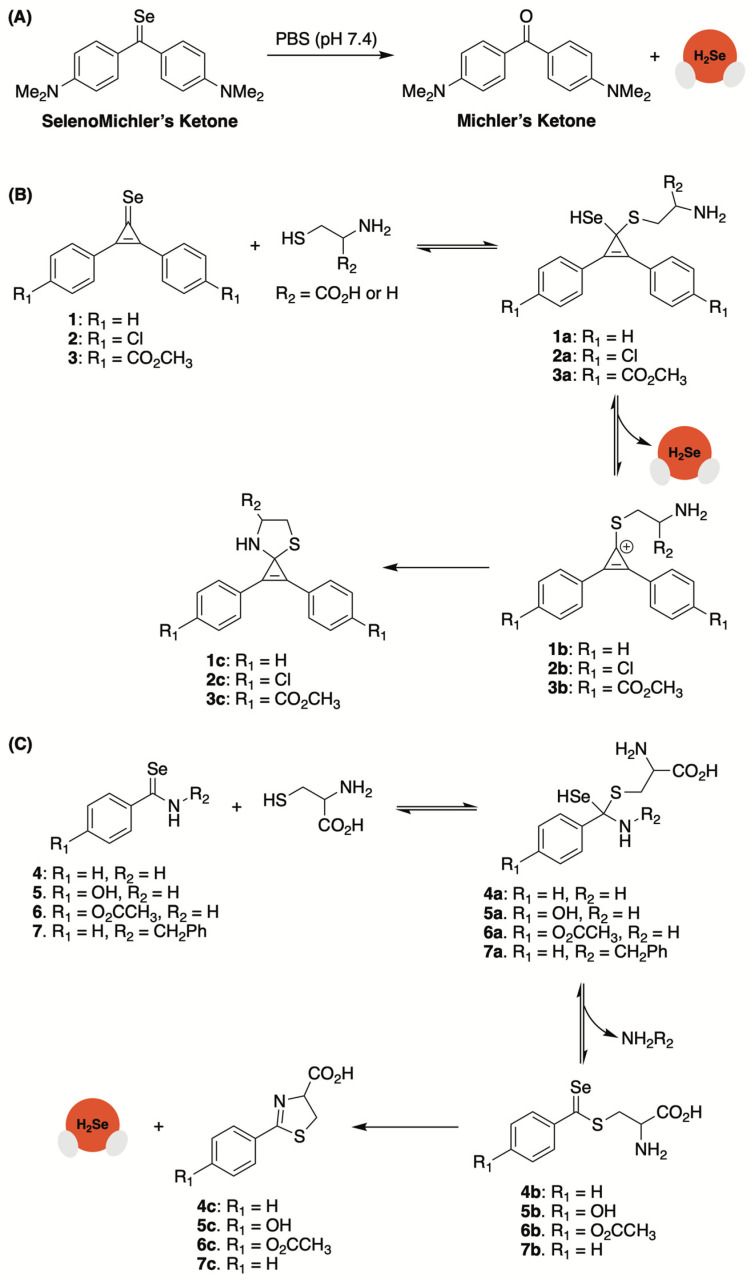
(**A**) Rapid hydrolysis of SelenoMichler’s ketone generates H_2_Se in PBS (pH 7.4). (**B**) Proposed mechanism for cysteine/thiol-triggered H_2_Se donation from selenocyclopropenones. (**C**) Proposed mechanism for cysteine-triggered H_2_Se donation from arylselenoamides.

Initially, the authors generated a selenium analogue of Michler’s ketone but found that it quickly hydrolyzed in a buffer, producing a red residue (Se^0^), presumably due to its rapid discharge of selenide (Figure 6A). Searching for a selenocarbonyl with a more tractable H_2_Se-releasing profile, selenocyclopropenones were then investigated. A small library was generated by treating the corresponding ketone with Woollins’ reagent (Figure 6B). Compound 1 was chosen for initial studies, evaluating its reactivity and selectivity as a selenide donor. HPLC analysis confirmed that 1 was stable in a 50% PBS/CH_3_CN (pH 7.4) mixture. The introduction of cysteine (2–10 mM), however, led to the expulsion of selenide and the subsequent formation of a red solid (Se^0^). This was further corroborated by an H_2_Se-selective gas detector. Donors 2 and 3, with electron-withdrawing substituents at the *para* position, were found to liberate even more H_2_Se than 1 in the presence of cysteine, implying that the rates of donation from this platform can be easily tuned via simple structural modifications that alter the electrophilicity of the selenocarbonyl. The reaction between 1 and cysteine was further scrutinized by high-resolution mass spectrometry (HRMS) and identifiable byproducts were uncovered. Based on these observations, a mechanism for cys/thiol-triggered H_2_Se release from selenocyclopropenones was put forward by the authors (Figure 6B).

Aryl thioamides were previously shown to function as an advantageous platform for the controlled delivery of H_2_S under biologically relevant conditions [69,70,71]. Yi and co-workers suspected that selenoanalogues would provide an avenue for H_2_Se donation under similar conditions (Figure 6C) [68]. To test their hypothesis, a small library of selenoamides was generated by treating benzamide derivatives with Woollins’ reagent or by subjecting 4-hydroxybenzonitrile to a mixture of selenium powder and NaBH_4_. Selenobenzamide (4) was shown to be stable in PBS (pH 7.4), but the addition of cysteine led to the formation of red Se^0^. This was further substantiated with an H_2_Se gas detector. As expected, the rate of H_2_Se release from 4 was found to be significantly slower than 1. However, it was noted that the selenoamide platform could be structurally modified to alter reaction kinetics. For example, the introduction of an electron-donating hydroxyl group at the *para* position on the phenyl ring (5, Figure 6C) appeared to amplify the speed of selenide release. However, when the hydroxyl group was converted to a methyl ester (6, Figure 6C), its donating efficiency diminished somewhat, even relative to 4. On the other hand, amide *N*-alkylation (7, Figure 6C) was shown to suppress the rate of selenide delivery even further. HPLC analysis and DFT calculations were used to establish a mechanism for cysteine-triggered H_2_Se donation (Figure 6C). This proposed pathway aligns with the observation that treatment of arylselenoamides with other thiols that lack a nucleophilic amine (i.e., *N*-acetylcysteine, glutathione, and β-mercaptoethanol) fail to generate H_2_Se.

### 2.6. Selenocarbamates

**Figure 7:**
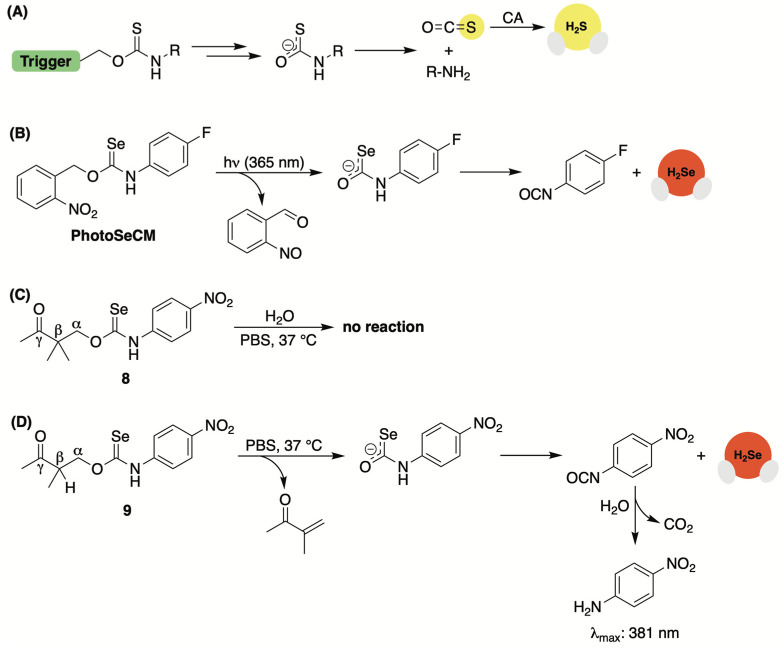
(**A**) General strategy for generating stimuli-responsive COS/H_2_S donors. (**B**) Proposed mechanism for the direct release of H_2_Se from PhotoSeCM upon irradiation at 365 nm. (**C**) Control compound used to highlight the stability of *γ*-ketoselenocarbamates in water. (**D**) Proposed mechanism for the direct release of H_2_Se from *γ*-ketoselenocarbamates containing a deprotonatable hydrogen at the β position.

Caged thiocarbamates have offered a reliable avenue for H_2_S delivery (Figure 7A). They also provide an opportunity to selectivity tune the release of H_2_S to a specific biological trigger (i.e., ROS [72,73,74,75,76], light [77,78], pH [79,80], and enzymes [81,82]). However, the production of H_2_S from this donor class is multi-layered, with the triggering event causing the donor to undergo a self-immolating process that first generates carbonyl sulfide (COS) prior to its quick conversation to H_2_S by the ubiquitous enzyme carbonic anhydrase (Figure 7A) [83]. 

Continuing with the common theme of prior H_2_S-releasing motifs laying the foundation for H_2_Se donor development, Pluth and co-workers were curious whether this framework could be reengineered to release COSe, providing a new means for the controlled delivery of selenide [84]. To test their hypothesis, the authors treated *p*-fluorophenyl isoselenocyanate with 2-nitrobenzyl alcohol in the presence of NaH to generate light-activated PhotoSeCM (Figure 7B). To analyze product formation upon photoactivation, the authors used ^19^F NMR to streamline the process. Using this method, consumption of PhotoSeCM (−117 ppm) was confirmed but the expected *p*-fluoroaniline product (due to COSe release) at −130 ppm was not observed. Instead, a signal matching *p*-fluoroisocyanate was detected, suggesting the direct liberation of selenide rather than COSe serving as an intermediary (Figure 7B).

Interested in assessing a second COSe/H_2_Se-releasing system but without the requirement of photoactivation to simplify the overall mechanism, Pluth and team also examined *γ*-ketoselenocarbamates, which were reconstituted from their earlier work on *γ*-ketothiocarbamates [80]. This H_2_S donor class undergoes an enol-mediated self-immolating process that delivers COS/H_2_S alongside *p*-nitroaniline, which can be used to track reaction progress due to its UV–Vis signature at 381 nm. *γ*-Ketoselenocarbamates were constructed from *p*-nitrophenyl isoselenocyanate and their activation at different pH values was monitored by UV–Vis. While compound 8, which lacks deprotonatable hydrogens at the β position, failed to generate *p*-nitroaniline (Figure 7C), compound 9, as expected, showed a pH-dependent rate of release, increasing at higher pH values (Figure 7D). In buffer, the authors were unable to provide direct experimental evidence of selenide release from this donor system as the ensuing *p*-nitrophenyl isocyanate byproduct is unstable and likely to hydrolyze quickly to *p*-nitroaniline and CO_2_. Nevertheless, computational studies corroborate the likelihood of direct selenide liberation as it was calculated to be by far the lowest energy decomposition pathway, underscoring the fundamental differences in thiocarbamate/selenocarbamate reactivity. 

### 2.7. γ-Ketoselenides

**Figure 8:**
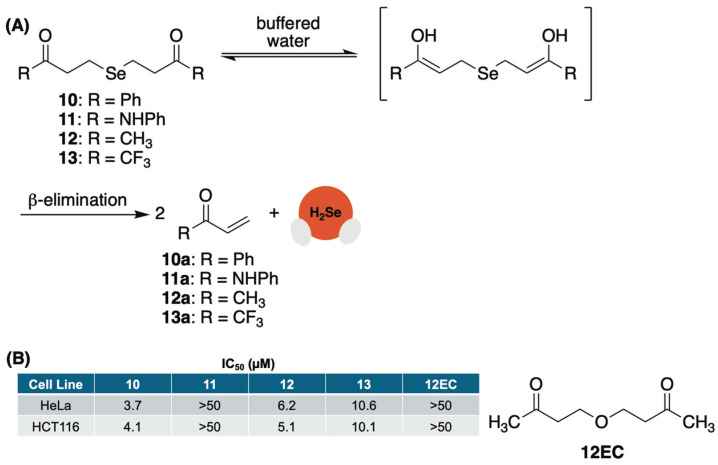
(**A**) A library of *γ*-ketoselenides that undergo base-promoted α-deprotonation/β-elimination to release H_2_Se. (**B**) Cell growth inhibition of HeLa and HCT116 cells in culture. IC_50_ values were determined after a 24 h incubation period with donor.

An analogous base-mediated selenide delivery system was first reported by us in 2022 [85]. In this study, we prepared a library of *γ*-ketoselenides (10–13, Figure 8A) upon treatment of the corresponding *γ*-ketohalide/tosylate with a solution of sodium selenide that was generated in situ by reducing elemental selenium with NaBH_4_. 

Once in hand, we first monitored the release of selenide from 10 (Figure 8A) in a 1:1 mixture of CD_3_CN and deuterated phosphate buffer (50 mM, pD 7.4) by tracking the formation of byproduct 10a (Figure 8A) using ^1^H NMR. During the experiment, we observed a decrease in the intensity of the protons alpha to selenium while the signal of the terminal enone protons simultaneously increased. The addition of 1,4-dioxane as an internal standard allowed us to track the concentration of both and confirm that the consumption of 10 correlated well with the production of 10a. During these studies, we also observed the formation of a red film in our NMR tube, which we attributed to the rapid oxidation of H_2_Se in an aqueous buffer and its conversion to Se^0^. This was confirmed by ^31^P NMR and the formation of triphenylphosphine selenide upon treatment with triphenylphosphine. 

Appearing to proceed through an α-deprotonation/β-elimination sequence, we suspected that an enhanced rate of H_2_Se donation from *γ*-ketoselenides would be observed at both higher pH values and with donors that possess more acidic alpha protons. To test our hypotheses, the same kinetic studies were repeated at pH 6 and 8.5. As anticipated, the rate of selenide donation was found to be intensified under more basic conditions (pH 8.5) and hindered under more acidic conditions (pH 6). Similarly, alpha proton acidity appeared to play a significant role in donor reactivity with 13 undergoing rapid selenide liberation (confirmed with trapping experiments using benzyl bromide), while 11 was found to be considerably more stable than both 10 and 12. In total, these experiments not only verified the release of selenide from *γ*-ketoselenides but also the mechanism through which it occurs.

The anticancer activity of these compounds was also assessed in HeLa (human cervical cancer) and HCT116 (human colon cancer) cells in culture (Figure 8B). Predictably, 11, which was found to be stable in buffer for months at a time, liberating only trace amounts of selenide, was found to be completely inactive in both cell lines. Conversely, 10, 12, and 13 exhibited low micromolar activity. As a key control, compound 12EC, an oxygen conger of 12, was also tested and found to be completely inactive, confirming that the activity of donors is due to their release of selenide and not the other components of the reaction. It is also worth mentioning that 11, which liberates selenide at an order of magnitude faster than both 10 and 12, was found to be two-fold less potent in both cell lines. This implies that greater antiproliferative activity may be achieved through continuous exposure to low levels of H_2_Se for a prolonged period as opposed to a rapid surge in selenide concentration that is likely afforded by 11. 

### 2.8. 5′-O-Selenophosphate Nucleosides

It had been previously shown by Kaczmarek and colleagues that 5′-*O*-thiophosphate nucleosides function as an H_2_S source in the presence of histidine triad nucleotide-binding protein 1 (HINT1) [86,87]. Consistent with the common theme throughout this review, Kaczmarek speculated that a selenium congener might function as a HINT1 substrate [88]. If so, then perhaps H_2_Se, like H_2_S, would be afforded as a byproduct of the enzyme’s hydrolase activity. 

**Figure 9:**
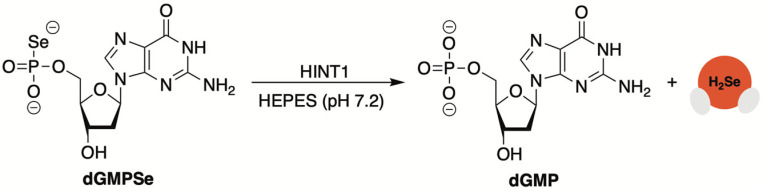
5′-*O*-Selenophosphates (**dGMPSe**) undergo HINT1-catalyzed hydrolysis to generate H_2_Se.

To test this premise, 2′-deoxyguanosine-5′-*O*-selenophosphate (dGMPSe) was produced using a known method for generating phosphoroselenoates [89]. After confirming the stability of dGMPSe in the buffer alone, its HINT1-catalyzed hydrolysis was assessed. This selenophosphate derivative was found to be a substrate for HINT1 with both deoxyguanosine monophosphate (dGMP), confirmed by HPLC, and H_2_Se, detected by fluorescence spectroscopy and the use of the reaction-based probe SF7 [90], were observed as products of the enzyme-catalyzed hydrolysis (Figure 9).

After confirming the HINT1-promoted release of H_2_Se in vitro, the cytotoxicity of dGMPSe was evaluated in HeLa cells. Compared to dGMP, which was found to be nontoxic at all concentrations tested, dGMPSe exhibited dose-dependent cytotoxicity with an IC_50_ value of 8 µM after a 24 h incubation period. Furthermore, dead cells were shown to exhibit higher fluorescence in the presence of SF7, further supporting the notion that released H_2_Se from dGMPSe is responsible for cell death. 

## 3. Chemical Tools for H_2_Se Detection

### 3.1. Nonspecific Electrophilic Traps: Dinitrofluorobenzene, Benzyl Bromide, and Iodoacetamide

While selective microsensors and a plethora of reaction-based fluorescent probes offer reliable methods for sulfide detection, analogous chemical tools with selenide specificity are lacking. Furthermore, the methylene blue assay, a ubiquitous method for sulfide quantification in buffer, is unlikely to translate to accurate selenide detection due to its rapid oxidation and overall chemical instability compared to that of sulfide. As such, many researchers have turned to the use of nonspecific electrophilic traps as a quick and dirty method for selenide sensing and validation of H_2_Se donor activation.

**Figure 10:**
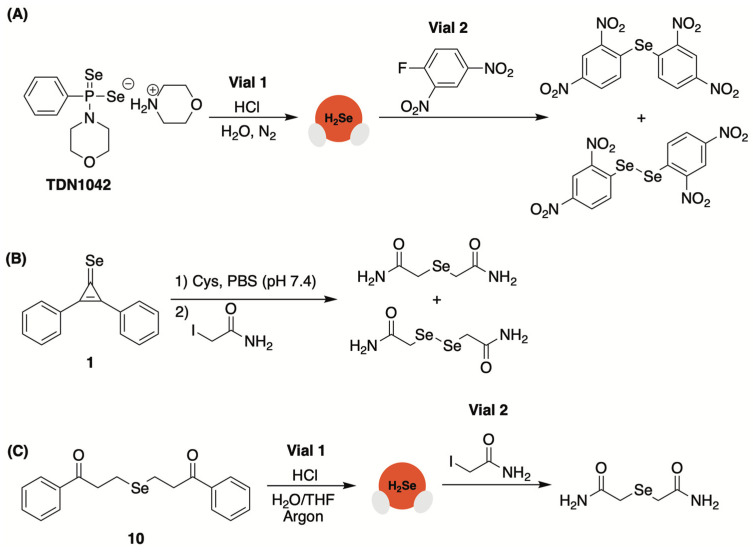
Use of nonspecific electrophilic traps to confirm H_2_Se release from donors ((**A**) TDN1042, (**B**) 1, (**C**) 10) by forming stable selenide/diselenide products that can be easily identified by spectroscopic methods.

As mentioned earlier, Pluth and co-workers relied on dinitrofluorobenzene (DNFB) for confirmation of H_2_Se donation from TDN1042 (Figure 10A) [65]. However, the high electrophilicity of DNFB, coupled with the augmented nucleophilic character of selenium, made this a challenging endeavor. The authors had previously observed the P=Se moiety of the donor reacting directly with other electrophiles, such as benzyl bromide, making it impossible to distinguish donor alkylation from the alkylation of H_2_Se. Thus, to unequivocally establish selenide release from TDN1042, the authors placed the DNFB trapping solution in a vial separate from the donor. Under this setup, when TDN1042 was acidified with HCl and sparged with N_2_, the liberated H_2_Se was volatilized into the headspace and bubbled through the separate trapping solution containing an excess of DNFB. The contents of the trapping solution were then analyzed by HPLC with the expected mono and diselenide products being clearly visible (Figure 10A). 

Likewise, Yi and colleagues first utilized iodoacetamide for quick confirmation of selenide donation from their selenocyclopropenone-based donors (Figure 10B) [68]. This analysis was further complicated by the fact that cysteine, which can be consumed by the added iodoacetamide, was used to trigger H_2_Se release from this donor class. Nevertheless, using HRMS the authors observed the corresponding mono and diselenide products, which is consistent with the release of selenide and the autooxidation process. 

We, too, relied on nonspecific electrophiles, both benzyl bromide and iodoacetamide, for confirmation of H_2_Se liberation from our *γ*-ketoselenide-based system [85]. Like the Pluth group, we also sought to unambiguously confirm H_2_Se donation from 10 by trapping the gas in a separate vial (Figure 10C). We elected to use iodoacetamide for these experiments as the ensuing product from its trapping of volatilized H_2_Se would be easily recognizable by HRMS. Indeed, when 10 was acidified and sparged with argon, released H_2_Se was transferred through a cannula needle and into a separate trapping solution where the expected selenide product was clearly visible by HRMS. 

### 3.2. Fluorescent Sensors Based on Benzoselenadiazole Se–N Bond Cleavage

**Figure 11:**
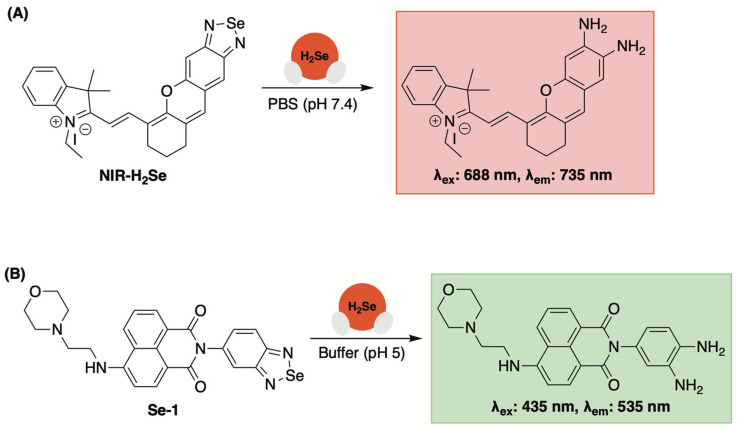
(**A**) Reaction-based fluorescent sensor with selectivity towards H_2_Se based on benzoselenadiazole Se–N bond cleavage. (**B**) A lysosomal-targeting fluorescent sensor for H_2_Se bioimaging.

A small molecule fluorescent probe with high selectivity towards H_2_Se was first reported by Tang and colleagues in 2016 (NIR-H_2_Se, Figure 11A) [91]. By fabricating a benzoselenadiazole moiety onto a mercaptan dye, the authors found that Se–N bond cleavage occurred quickly in the presence of H_2_Se, but not when exposed to other selenols or thiols, to afford to a fluorescent diamine reporter (λ_ex_: 688 nm, λ_em_: 735 nm).

With an H_2_Se-selective sensor in hand, the authors then used NIR-H_2_Se to monitor cellular H_2_Se levels in HepG2 cells using Na_2_SeO_3_ as a metabolic precursor. In this experiment, a dose and time-dependent increase in fluorescence was observed, as expected. Moreover, these investigations were conducted under both hypoxic (1% pO_2_) and normoxic (20% O_2_) conditions, with a significant reduction in fluorescence being observed during the latter. This was attributed to rapid H_2_Se oxidation and the production of superoxide and other reactive oxygen species while exposed to higher O_2_ levels. Based on these observations, it was concluded that the anticancer activity of Na_2_SeO_3_ in HepG2 cells under normoxic conditions can be attributed to ROS-induced cell death. Conversely, under hypoxic conditions, which is a hallmark of solid tumors, a non-oxidative stress mechanism is likely in play due to a notable buildup in cellular H_2_Se. This hypothesis was further validated in a solid tumor mouse model using NIR-H_2_Se. 

A lysosomal-specific H_2_Se sensor based on this same framework was reported by Zhang and Jing in 2019 (Se-1, Figure 11B) [92]. In addition to a benzoselenadiazole moiety for selective H_2_Se detection, a morpholino group was appended to the sensor for lysosomal-targeting [93]. Under simulated lysosomal conditions (acetate buffer, pH 5), a notable increase in fluorescence at 535 nm (λ_ex_: 435 nm) was observed when a solution of Se-1 was exposed to H_2_Se (but not other biologically relevant analytes). Moreover, the fluorescent enhancement of Se-1 in the presence of H_2_Se was found to be consistent within a pH range of 4.5–7, confirming its compatibility with lysosomal conditions. 

After establishing its reactivity and photophysical properties in buffer, lysosomal-targeting of Se-1 was confirmed in HepG2 cells using LysoTracker Blue (Pearson’s correlation coefficient of 0.91). Additionally, using Se-1, the authors observed elevated levels of lysosomal H_2_Se in hypoxic HepG2 cells, whereas little lysosomal fluorescence was observed under normoxic conditions. 

To date, benzoselenadiazoles have not been used as a recognition subunit to validate selenide delivery from novel donor scaffolds. However, its reported high reactivity towards H_2_Se could prove useful in future studies as the transient nature of H_2_Se requires rapid detection for accurate monitoring. 

### 3.3. Fluorescent Sensors Based on Disulfide Bond Cleavage

**Figure 12:**
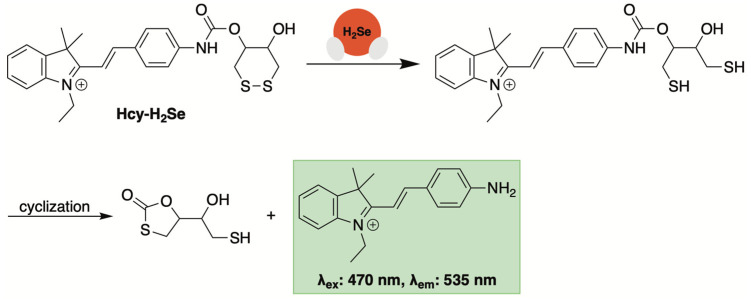
Proposed mechanism for Hcy-H_2_Se turn-on fluorescence initiated by H_2_Se-promoted disulfide reduction.

A second-generation sensor from Tang and co-workers employed disulfide bond reduction as a recognition mechanism for H_2_Se-initiated turn-on fluorescence (Hcy-H_2_Se, Figure 12) [94]. Thiol-activated prodrugs and chemosensors that utilize disulfide bond reduction as their initiation step have been widely reported [95]. Based on these accounts, it was suspected that a more stable cyclic disulfide (i.e., a six-membered ring) would respond much more quickly to H_2_Se, given its heightened nucleophilicity, thereby imparting selectivity over other selenols and thiols.

Hcy-H_2_Se was generated by combining oxidized dithiothreitol [96] with a masked hemicyanine dye via a carbamate linker. When exposed to H_2_Se, a maximum fluorescence intensity (λ_ex_: 470 nm, λ_em_: 535 nm) was reached almost immediately, indicating rapid H_2_Se-initiated disulfide bond reduction and cyclization (Figure 12). Selectivity studies indicated that the addition of glutathione, H_2_S, selenocysteine, dithiothreitol, bovine serum albumin, and thioredoxin reductase yielded little fluorescence compared to H_2_Se. Moreover, the emission intensity at 535 nm correlated well with increasing concentrations of H_2_Se, affording a good linear relationship between the two. 

Bioimaging of H_2_Se in live human cells was accomplished with Hcy-H_2_Se [94]. HepG2 cells exposed to Na_2_SeO_3_ and Hcy-H_2_Se under hypoxic conditions exhibited fluorescence that was both dose- and time-dependent. The authors also demonstrated that hypoxic tumor regions in mice injected with sodium selenite could be imaged with Hcy-H_2_Se. This further supports the anticancer effects of selenium in hypoxic solid tumors being due to an increase in reductive rather an oxidative stress. 

### 3.4. Spectroscopic Sensors Based on Nucleophilic Substitution 

**Figure 13:**
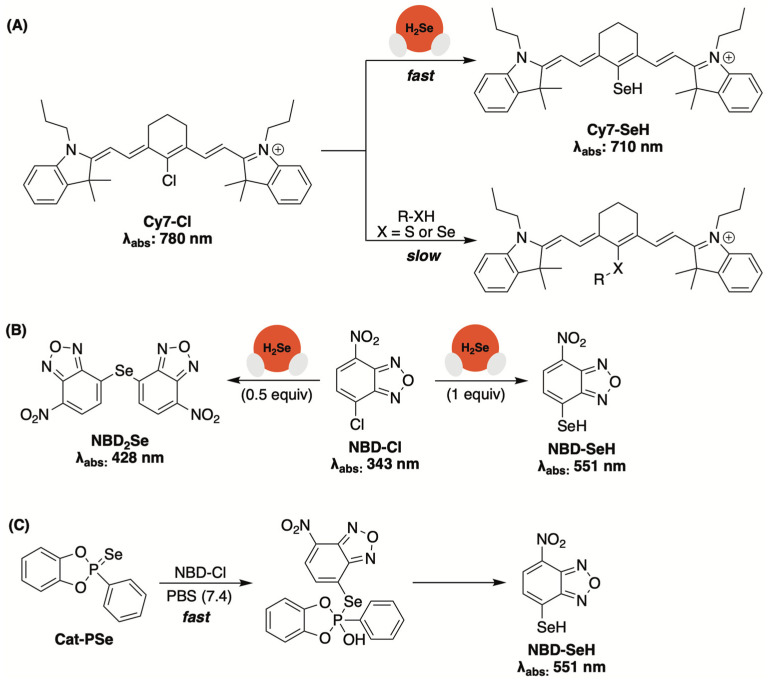
(**A**,**B**) Commercially available electrophilic traps that provide a colorimetric readout for monitoring H_2_Se donor progress. (**C**) Cautioning researchers that complementary H_2_Se measurements should be employed to avoid confusing donor alkylation with the alkylation of released H_2_Se in solution.

While examining selenocyclopropenones and selenoamides as potential cysteine-activated donors (Figure 6), Yi and collaborators developed a quantitative assay for H_2_Se based on its nucleophilic substitution with a carefully chosen electrophilic species [68]. Upon testing several potential candidates, the authors discovered that commercially available Cy7-Cl reacted much more quickly with selenide than other selenols and thiols, to form Cy7-SeH (Figure 13A). As the reaction progressed, it was noted that the starting absorbance of 780 nm (Cy7-Cl) was shifted to 710 nm (Cy7-SeH), offering a convenient colorimetric method for monitoring reaction progress. The reaction between Cy7-Cl (500 µM) and various amounts of Na_2_Se (25–350 µM) was also monitored HPLC, with a plot of Cy7-SeH peak area vs. selenide concentration yielding a straight line. This calibration curve was then used to effectively determine the H_2_Se-releasing efficiency from selenocyclopropenones in the presence of cysteine. 

While evaluating the H_2_Se-releasing efficiencies of cyclic-PSe donors (Figure 5), the Pluth group found that commercially available 4-chloro-7-nitrobenzofurazan (NBD-Cl), which had been previously used to detect H_2_S [97], could also be used to trap selenide via a nucleophilic aromatic substitution reaction (Figure 13B) [66]. Initially, the authors analyzed the reaction between NBD-Cl and tetrabutylammonium hydroselenide (NBu_4_SeH) in PBS (pH 7.4). They found that substoichiometric amounts of selenide provided NBD_2_Se (λ_abs_: 428 nm), while stoichiometric HSe^–^ yielded NBD-SeH as the primary product (λ_abs_: 551 nm). With confirmation of a colorimetric response, NBD-Cl was then used to monitor the hydrolysis of Cat-PSe in PBS (Figure 13C). While the formation of NBD-SeH was clearly visible (λ_abs_: 551 nm), the rate of apparent H_2_Se liberation appeared to occur much faster when compared to earlier ^31^P NMR hydrolysis experiments. The authors attributed this notable rate enhancement to donor alkylation, rather than hydrolysis, while in the presence of a strong electrophile, such as NBD-Cl. Therefore, while the use of NBD-Cl appears to provide a convenient colorimetric method for monitoring donor progress, additional trapping experiments are likely necessary to untangle donor alkylation from the alkylation of released H_2_Se in solution. 

### 3.5. Fluorescent Sensors Based on Azide Reduction

**Figure 14:**
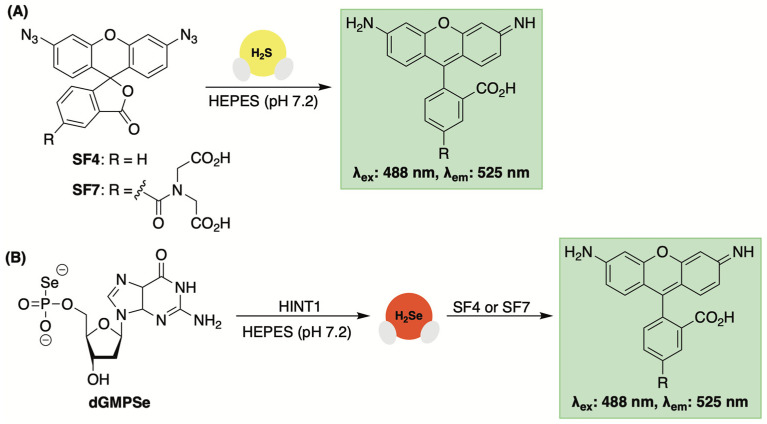
(**A**) Aryl azide reduction for H_2_S-initiated turn-on fluorescence. (**B**) The use of aryl azides as fluorescent probes for tracking H_2_Se liberation from donor compounds.

Like H_2_S-responsive NBD-Cl being reintroduced for H_2_Se detection, H_2_S-sensitive fluorescent sensors that rely on aryl azide reduction were reexamined for their capacity to detect H_2_Se [89]. Probes SF4 and SF7 (Figure 14) were previously engineered by Chang and co-workers for monitoring H_2_S levels under physiological conditions [90,98]. While stable towards other sulfur-containing molecules, H_2_S initiates aryl azide reduction on the masked rhodamine dye, resulting in a turn-fluorescent response (Figure 14A). 

Given the increased reactivity of H_2_Se, Kaczmarek and co-workers suspected that these same sensors would be responsive towards H_2_Se and used them to confirm selenide release from dGMPSe in both in a buffer and in live HeLa cells (Figure 14B) [88]. In fact, when using SF7 and SF4, higher fluorescence values were observed with dGMPSe compared to its sulfur congener, which likely stems from the higher reactivity and reductive properties of H_2_Se compared to that of H_2_S. Thus, while not selective for H_2_Se, aryl azide reduction appears to be a potential method for the real-time monitoring of donor progress.

## 4. Conclusions and Outlook

The continued evolution of H_2_Se-specific chemical tools is a prime objective for those interested in uncovering the (patho)physiological effects of hydrogen selenide. While donor compounds capable of providing the slow and sustained release of H_2_Se have begun to emerge, the introduction of additional stimulus-responsive donors (i.e., enzyme or bioanalyte-triggered) with greater spatiotemporal control over their biological delivery of H_2_Se will serve as a great advancement in the field. H_2_Se detection and quantification, especially when liberated from a donor scaffold, remains a great challenge. Most current methods rely on electrophiles to trap the released selenide, which often leads to interference from the donor itself given the intensified nucleophilic character of selenium. New approaches for detection that avoid the introduction of strong electrophiles should continue to be explored, including additional reaction-based fluorescent sensors that can be used in cellular and in vivo imaging experiments. Together, these compounds will continue to unearth the unique aspects of H_2_Se chemical biology, while providing new evidence that further supports (or refutes) the addition of H_2_Se as the fourth gasotransmitter.

## Data Availability

Not applicable.
